# Using Vis-NIR Spectroscopy for Predicting Quality Compounds in Foods

**DOI:** 10.3390/s22134845

**Published:** 2022-06-27

**Authors:** Mercedes del Río Celestino, Rafael Font

**Affiliations:** Agri-Food Laboratory, CAGPDS, Avd. Menéndez Pidal, s/n, 14080 Córdoba, Spain; rafaelm.font@juntadeandalucia.es

Over the past four decades, near-infrared reflectance spectroscopy (NIRS) has become one of the most attractive and used technique for analysis as it allows for fast and simultaneous qualitative and quantitative characterization of a wide variety of food samples [1]. NIR spectroscopy is also essential in various other fields, e.g., pharmaceuticals [2], petrochemicals [3], textiles [4], cosmetics [5], medical applications [6], and chemicals such as polymers [7].

The high level of interest in NIR spectroscopy among scientific and professional sectors demonstrates its relevance. We hope that this Special Issue’s scope facilitates the interchange of ideas and thereby aids in expanding the frontiers of this field of knowledge. Furthermore, we aim to provide readers with a comprehensive summary of present state-of-the-art NIR spectroscopy, trends in development, and future possibilities. We believe that by doing so, we will be able to provide a chance for all contributors to make their results and methodologies more visible, as well as to highlight current achievements in their respective fields made possible by the use of NIR spectroscopy.

This Special Issue has had a resoundingly enthusiastic response, with several submissions from academics and professional spectroscopists, resulting in a collection of 13 papers, including one exhaustive review paper [8,9,10,11,12,13,14,15,16,17,18,19,20]. The articles submitted represent the variety of the discussed field well, covering a wide range of topics related to NIR spectroscopy. The majority of the papers concentrate on applied qualitative and quantitative analysis in a variety of fields.

New progress has been made in improving food quality thanks to the first investigation. Accordingly, it was determined that the use of variable selection algorithms provided a better performance in predicting the amount of organophosphorus pesticide residues in tomatoes using NIRS than the use of all spectral data [8].

The feasibility of measuring physicochemical quality parameters of mangetout pods by means of VIS-NIRS has also been demonstrated. The results revealed that the models allow for an accurate quantification of protein and total polyphenol content and a rough screening method of the samples for color parameters (c* and h*), firmness, ascorbic acid content and pH [9].

In addition, despite the advantages of NIR nondestructive measurement, there is a lack of basic studies comparatively evaluating various forms of sampling with and without minimal processing. The analyses conducted in this Special Issue have showed that Vis-NIR spectroscopy could be used as a quick method to assess the abundance of chemical compounds (soluble solids content, saccharose (Pol), fiber, Pol of cane, and total recoverable sugars) of sugarcane. Moreover, the performance of the models on defibrated cane and raw juice samples were similar, but defibrated cane samples involve less preparation as they do not require juice extraction [10].

For the first time, this research shows the applicability of NIR spectroscopy to assess volatile phenol contents (guaiacol, 4-methyl-guaiacol, eugenol, syringol 4-methyl-syringol and 4-allyl-syringol) and confirms the ability of this technique to quantify compounds that contribute to the sensory quality of aged wine spirits [11].

NIRS technology can be a powerful tool to ensure the quality of food products and prevent fraud. From the results obtained, it can be concluded that NIRS together with artificial neural networks allow for the accurate prediction of almost all sensory parameters selected for an exhaustive characterization of dry-cured beef meat—cecina—quality. It would be possible to substitute the sensory panel with a faster, reliable, nondestructive and cheaper instrumental technique that may be implemented on site [12].

In addition, this Special Issue showed that NIRS is a feasible and useful tool for screening purposes, and it has the potential to predict most of the fatty acids of freeze-dried beef [13].

Moreover, a comprehensive review of the state of the art in research and the actual potential of NIRS for the analysis of olive oil has been included. It can be concluded that the four most common physicochemical parameters that define the quality of olive oils, namely free acidity, peroxide value, K232, and K270, can be measured using NIRS with high precision. In addition, NIRS is suitable for the nutritional labeling of olive oil because of its great performance in predicting the total fat, total saturated fatty acid, monounsaturated fatty acid, and polyunsaturated fatty acid contents in olive oils [14].

Likewise, the potential of hyperspectral imaging can be also recognized on the basis of the articles collected in this Special Issue [15,16,17]. Hyperspectral imaging (his) emerges as a non-destructive and rapid analytical tool for assessing food quality, safety, and authenticity. This technology can not only identify the physical chemistry characteristics of a substance through spectroscopic analysis, but also simultaneously obtains information about the spatial distribution of certain components through image analysis [21]. In this Special Issue, we present the possibility of rapidly inspecting and detecting *Escherichia coli* and *Salmonella typhimurium* on the surface of food processing facilities, which is a major global public health problem [22], via fluorescence hyperspectral imaging and various discriminant analysis techniques [15].

This Special Issue aims to investigate the potential of combining the spectral and spatial features of HSI data with the aid of deep-learning approaches for the pixel-wise classification of food products (sweet products and salmon fillets). The results demonstrated that spectral pre-processing techniques prior to convolutional neural network model’s development can enhance the classification performance. This work will open the door for more research in the area of practical applications in food industry [16].

Important information is generated for the agrifood industry thanks to the new data provided in this Special Issue. Hyperspectral imaging technology has been used to develop a method for diagnosing the soil plant analysis development (SPAD) value and mapping the spatial distribution of chlorophyll in leaves located at different positions during the growth season of pepper plants. The results show that hyperspectral imaging is a very promising technology and has great potential for the intuitive monitoring of crop growth, laying the foundation for the development of hyperspectral field dynamic monitoring sensors [17].

The growing applicability and importance of portable NIR spectrometers is reflected by several articles, opening a new window for the utilization of these types of instruments in the analysis and monitoring of the composition of foods. In this context, the ability of a micro-near-infrared portable instrument to predict vitamin C in both whole and pureed Kakadu plum fruit samples was demonstrated [18].

In this regard, the use of MicroNIR as a tool for estimating dry matter and reducing sugars of fresh potato in a warehouses by directly measuring the tubers without chemical treatment and destruction of samples has been demonstrated. The efficiency of such automation techniques optimizes the management of industrial processing, guaranteeing the quality of the potato tubers during in-line processing [19].

In this work, we also focused on the development of a real-time and simple methodology to quantify the macronutrients (fat, raw protein and carbohydrates) in breast milk using a portable NIRS instrument. Notably, the implementation of this procedure requires the use of low-cost and handheld NIRS instruments where expert personnel are not required for analyzing samples, facilitating the quality-control procedure in the feeding of newborns in neonatology units [20].

It should be noted that these contributions accurately reflect the diversity and dynamism of current NIR spectroscopy development trends.

This Special Issue is accessible through the following link: https://www.mdpi.com/journal/sensors/special_issues/NIR-Foods (accessed on 24 June 2022). We would like to thank all of the authors and co-authors for their contributions, as well as all of the reviewers for their time and effort in carefully analyzing the submissions. Last but not least, we would like to express our gratitude to the editorial office of *Sensors* for their cooperation in preparing this Special Issue.
